# From plant genomes to protein families: computational tools

**DOI:** 10.5936/csbj.201307001

**Published:** 2013-08-14

**Authors:** Manuel Martinez

**Affiliations:** a Centro de Biotecnología y Genómica de Plantas (UPM-INIA), Campus Montegancedo, Universidad Politécnica de Madrid, Autovía M40 (Km 38), 28223-Pozuelo de Alarcón, Madrid, Spain

**Keywords:** Bioinformatic tools, Genomic databases, Genome annotation, Plant genome sequences, Plant protein families, Clustering methods

## Abstract

The development of new high-throughput sequencing technologies has increased dramatically the number of successful genomic projects. Thus, draft genomic sequences of more than 60 plant species are currently available. Suitable bioinformatics tools are being developed to assemble, annotate and analyze the enormous number of sequences produced. In this context, specific plant comparative genomic databases are become powerful tools for gene family annotation in plant clades. In this mini-review, the current state-of-art of genomic projects is glossed. Besides, the computational tools developed to compare genomic data are compiled.

## 1. Plant genome projects

The access to the primary DNA sequence has become a fundamental resource in biology. Recent advances in sequencing technologies and associated bioinformatic and computational tools have led to a deep increase in our knowledge of plant genomes [1–3].

**Figure 1:**
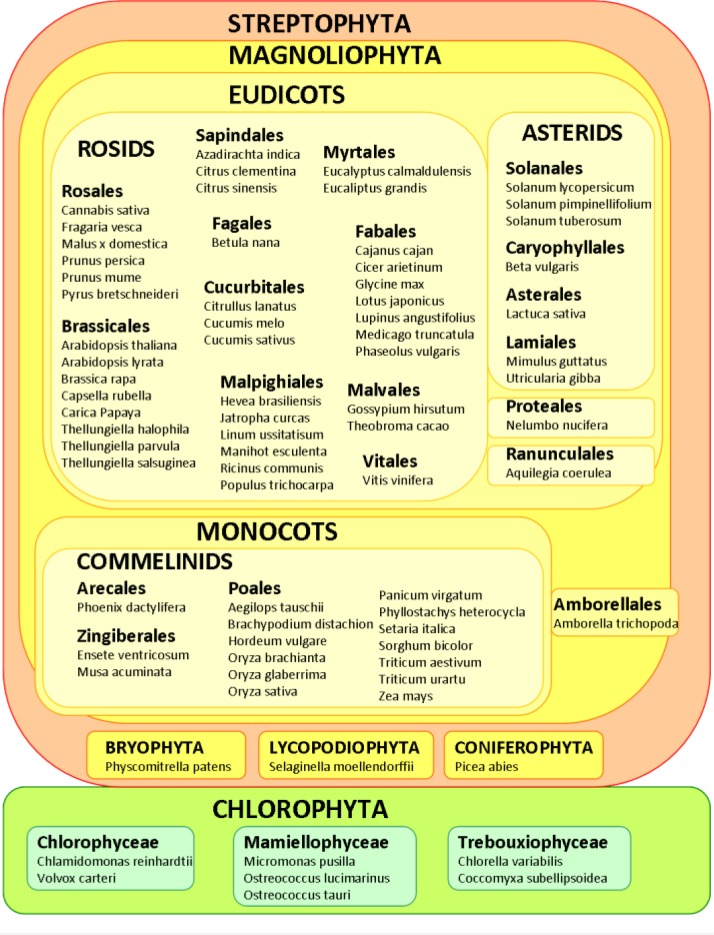
State-of-art of plant genome sequencing projects with available sequences.

According to the Genomes On-Line Database (GOLD), more than 20 plant genomes have been already completed and there are more than 200 ongoing plant genomic projects. Searches at the NCBI genomes database increase the number of species with draft DNA nuclear genomic sequences to more than sixty. Information of about 50 species of land plants with draft genome sequences is compiled in the CoGepedia web page (Table 1). The genomes of the eudicot model plant for plant biology, *Arabidopsis thaliana*, and the monocot crop model plant rice (*Oryza sativa*) were the first genomes to be sequenced. Nowadays, several other plant species from both, eudicot and monocot clades have been completely sequenced and their sequences are publicly available (Figure 1). Among eudicot species, there are examples from the most important orders included in the subclasses Rosids and Asterids, as well as the genome of the basal eudicot *Amborella trichopoda*. In contrast, due to their global agronomical value, most sequenced monocots species belongs to the Poales order. Besides, great efforts have been made to sequence basal plant species to deal with evolutionary challenges. Several algae genomes belonging to the main algal orders, a moss, *Physcomitrella patens*, and a pseudofern, *Selaginella moellendorffii* have been completely sequenced. Besides, technology advancements have now made feasible the sequencing of the extremely large conifer genomes, and very recently the first gymnosperm genome has been sequenced and published [4].

**Table 1:** **Table 1**. Several bioinformatics tools for plant genome analyses.

Bioinformatic tool	URL
*Plant genome Databases*	
Genomes on-line database	http://www.genomesonline.org/cgi-bin/GOLD/index.cgi
NCBI genomes	http://www.ncbi.nlm.nih.gov/genome
CoGePedia	http://genomevolution.org/wiki/index.php/Sequenced_plant_genomes
*Plant Comparative Genomic Databases*	
Gramene	http://www.gramene.org/
PlantGDB	http://www.plantgdb.org/
MIPS PlantsDB	http://mips.helmholtz-muenchen.de/plant/genomes.jsp
EnsemblPlants	http://plants.ensembl.org/index.html
*Plant Comparative Genomic Databases/Protein family databases*	
PLAZA 2.5	http://bioinformatics.psb.ugent.be/plaza/
Phytozome 9.1	http://www.phytozome.net/
GreenPhylDB 3.0	http://www.greenphyl.org/v2/cgi-bin/index.cgi

Main genome centers, such as JGI (Joint Genome Institute), BGI (Beijing Genomics Institute), JCVI (J. Craig Venter Institute) or MSU (Michigan State University), support most of the completed or ongoing plant genomic projects. Sequencing technologies for plant genomics research have quickly evolved through the last years [5]. Formerly, plant genomes have been usually sequenced by the Sanger sequencing technology. The recent development of next-generation sequencing (NGS) technologies that offer improvements in throughput and cost efficiency by using massively parallel sequencing systems has lead to their use in plant genomic sequencing. Then, most of the genomes sequenced in the last years, such as that of the cucumber, the cassava, the cacao or the strawberry have been mostly based on Illumina, Roche 454 or SOLiD reads. This technology has also made possible the sequencing of large and complex genomes from several crop plant species, such as the hexaploid genome of the bread wheat. Promising third generation sequencing technologies, such as the Single-Molecule Real-Time Sequencer from Pacific Biosciences, the Heliscope Single Molecule Sequencer and the Ion Personal Genome Machine are becoming available of generating long sequence reads in a short time and at even lower costs per instrument run, which will increase the feasibility of complete complex plant genomic projects.

## 2. Assembly and annotation of plant genomes

Once a genome has been sequenced, its partial sequences must be assembled. Usually, plants are polyploids and have high rates of heterozygosity and repeats, with a great number of repetitive transposable elements. These challenging features of plant genomes are translated to large primary genome sequences [6, 7]. Then, *de novo* assembling of a typical plant genome is a very complex task that commonly creates a highly fragmented result, such as the case of the low quality genome assembly of *Phoenix dactylifera* with more scaffolds than gene models. Associated to the development of sequencing high throughput technologies, a series of bioinformatic and computational resources for genome assembly have been developed. In the last years, extensive work has been carried out to create enhanced computational resources for sequence compression and computation distribution, and for producing high-quality assemblies from short reads. In the Sanger sequencing era, genome assembly was usually performed at a genome center using an expensive common computational infrastructure. In the last decade, there have been numerous advances in computer technology and bioinformatics tools, such as the development of computer and grid clusters or of cloud computing, that now put access to compute resources within the practical reach of a single investigator [2, 8]. However, to overcome the high diversity, heterozygosity, ploidy, and repetitive nature of plant genomes a plant-specific genome assembler is still needed [6, 7].

The next step is to annotate the genome. Generally, genome-wide annotation of gene structures is divided into two distinct phases. The first phase is the computation phase, in which ab initio and /or evidence-driven gene predictions are generated and experimental transcript and protein sequences are aligned to the genomic sequence. The second phase is the annotation phase, in which the above data are synthesized into gene annotations [9]. How accurately a genome is annotated is an important issue to be controlled. Automated genome prediction pipelines rarely exceed accuracies of 80% at the exon level; meaning that a significant number of gene annotations contain any mis-annotated exon [10]. Highly fragmented assemblies are an additional feature that complicates a correct prediction of plant gene sequences. To improve the annotation accuracy, extensive efforts in increasing the amount of transcriptomic data should be made. Besides, manual annotators should review the evidence for each gene in order to decide on their intron-exon structures. However, it is so labour-intensive that most plant genome projects are mostly based on automated methods. To facilitate manual annotation, online genome-annotation tools are being developed, such is the case of ORCAE, a tool to do manual curation in a wiki-style, community based approach [11].

## 3. Plant genome databases

When the genomic sequence has been parsed, assembled and annotated, the corresponding data have to be compiled in a database to facilitate their management by scientific researches. Available plant genome databases have arisen around specific plant species or distinct plant clades. Some examples for single genome databases are The Arabidopsis Initiative Resource, TAIR (http://www.arabidopsis.org/) or The Rice Genome Annotation Project (http://rice.plantbiology.msu.edu/), and for plant clades are SGN for Solanaceae (http://solgenomics.net/) or GDR for Rosaceae (http://www.rosaceae.org/). Typically, these databases and associated web portals provide a uniform set of tools to analyze the genomic sequences they host, such as BLAST tools for sequence searching and GBrowse tools for genome visualization.

Around these primary genome databases several comparative genome databases have grown up as a framework for comparative plant genome research. These databases comprise database instances for different sequenced plants, and differ in the plant species they host, as well as in the variety of tools and resources they have. Some examples of comparative genome databases are Gramene [12], PlantGDB [13], MIPS PlantsDB [14], EnsemblPlants [15]; PLAZA [16]; GreenPhylDB [17], and Phytozome [18] (Table 1).

**Gramene** is an extension of the RiceGenes project and, although formerly focused in grasses, is now a resource for several major model and crop plants. Gramene includes a wide array of potentially useful data sets such as quantitative trait loci, metabolic pathways, genetic diversity, genes, proteins, germplasms, literature, ontologies and a fully-structured markers and sequences database integrated with genome browsers and maps.

**PlantGDB** provides access to sequence data of a great number of plant species as well as to a variety of sequence and genome analysis tools. As a major tool, PlantGDB provides annotated transcript assemblies for more than 100 plant species, with transcripts mapped to their cognate genomic context where available at genome browser graphical interfaces. PlantGDB also hosts a plant genomics research outreach portal that facilitates access to a large number of resources for research and training.

**MIPS PlantsDB** hosts individual databases for several crop and model plants and provides tools to visualize and investigate syntenic relationships between plant species, to transfer data from model systems to crops and to explore similarities and peculiarities of different plant species.

**EnsemblPlants** is a part of Ensembl Genomes, an integrative resource for genome-scale data from non-vertebrate species. EnsemblPlants provides a set of resources that includes reference sequences, gene models, transcriptional data, polymorphisms and comparative analysis for a great number of sequenced plants.

**PLAZA, Phytozome** and **GreenPhylDB** are also databases that host common tools for comparative genomic studies. However, their scope is more related with the analysis of protein families and will be analysed in the next section.

## 4. Classification of plant protein-coding genes into families

The annotation of sequenced genomes provides us a great number of putative protein-coding sequences. In the sequenced land plants, they rank from about 25,000 genes in several diploid species to more than 90,000 putative genes in bread wheat. Classification of protein-coding genes into families is crucial to understand functional genomics and is based on the structure, function and evolution of the proteins they encode [19]. Then, gene families can be defined as sets of evolutionary related genes shared by a number of different species and with often similar biological functions, or by a set of homologous genes within one species. Some gene families appear to be more dynamic during evolution and show species-specific gene members. Others are more conserved and consist of genes sharing common ancestry that have diverged by speciation (orthologous genes). Orthologous genes are particularly useful for the characterization of unannotated proteins by identifying annotated counterparts that share high sequence identity. Traditionally, gene families have been discovered using alignments of multiple sequences to detect specific residues or motifs conserved among a set of homologous proteins. This approximation has lead to the development of traditional signature databases, such as Pfam (http://pfam.sanger.ac.uk/), since these motifs (or signatures) have been shown to be important for protein functionality and are able to define a family of proteins. These databases use sequences obtained from prokaryotic and eukaryotic species, without focusing on plants, and are useful for annotating proteins based on amino acid sequence similarities.

Recently, novel bioinformatics tools have been developed for the analysis of gene families based on comparative genomics [19]. These tools have been integrated in comparative genomic databases that can be used to perform evolutionary and comparative analyses, and to study gene families and genome organization. Based on orthologous genes, comparative genomics provides a powerful approach to translate functional information from model species to crops. The most comprehensive comparative genomic databases that focus on plant gene families are PLAZA, GreenPhylDB and Phytozome [16–18]. These databases differ in the genomes they include and are based in new clustering techniques. New clustering methods are based on pairwise comparison of full-length protein sequences from BLAST searches and their objective is to find and group homologous sequences from a pool using different algorithms. These methods are powerful tools to classify many sequences rapidly, in an automated manner, and with reasonable accuracy, and have allowed discovering novel gene families not covered by signature methods. The plant comparative genomic databases are the best choice for the identification of members of a protein family in different species, which is particularly interesting for phylogenetic analyses and the prediction of gene function. The accuracy of the data retrieved from these databases was previously evaluated [19].

### GreenPhylDB database

The version GreenPhylDB 3.0 hosts 22 species of the *plantae* kingdom including one rodophyte (red *algae*), two chlorophytes (green *algae*), one moss, one lycopod, six monocots, and 11 eudicot species. From the annotated sequences of these genomes, almost 500,000 sequences were clustered using TribeMCL [20]. This software uses a Markov cluster algorithm for grouping proteins into families based on a pre-computed sequence pairwise similarity matrix. The pairwise similarity matrix was obtained by running Protein-Protein BLAST, and different levels of clustering (1 to 4) were achieved using increasing stringent thresholds. Then, GreenPhylDB clusters were annotated gathering high-quality information from cross reference databases and phylogenetic analyses of validated families were performed to infer orthologs and paralogs identification. Currently, GreenPhylDB database contains 7,095 clusters with more than 5 sequences at level 1. For each gene cluster, GreenPhylDB offers an easy access to gene composition of each gene cluster, providing protein domains, publications, external links and orthologous gene predictions.

### PLAZA database

The current PLAZA 2.5 version hosts 25 plant species covering a broad taxonomic range, including 13 eudicots, five monocots, one lycopod, one moss, and five algae. From the more than 900,000 genes annotated in these genomes, the 32,294 gene families present in PLAZA were delineated by first computing the protein sequence similarity through an all-against-all BLAST and then by applying graph-based clustering methods implemented in TribeMCL and OrthoMCL [21] to cluster genes in families and subfamilies. Available data in this database consist of structural and functional gene annotations, homologous gene families, multiple sequence alignments, phylogenetic inferences to identify biologically relevant duplication and speciation events, and collinear regions within and between species. The current version has developed a new Integrative Orthology Viewer that combines information from different orthology prediction methodologies to efficiently investigate complex orthology relationships.

### Phytozome database

Phytozome is a joint project of the Department of Energy's Joint Genome Institute and the Center for Integrative Genomics to facilitate comparative genomic studies amongst green plants. As of release v9.1, Phytozome provides access to thirty-one sequenced and annotated green plant genomes, including six algae, one moss, one lycopod, six monocots, and 27 eudicot species, which have been clustered into gene families at ten evolutionarily significant nodes. Gene families are constructed from an all-versus-all BLASTP alignment used to compute the evolutionary distance between each two proteins, the identification of orthologs via Reciprocal Best Hit or synteny analysis, and the accretion of paralogs using outgroup scores. The end result is a set of gene families defined across a series of evolutionary nodes. Phytozome provides a view of the evolutionary history of every plant gene at the level of sequence, gene structure, gene family and genome organization, while at the same time providing access to the sequences and functional annotations of the plant genomes it hosts.

As an alternative, gene families can be built from transcriptomic data. When a reference genome is available, transcriptome sequencing can provide evidence for gene model predictions and for the completeness and accuracy of a genome assembly. For species lacking a genome sequence, transcript assembly can be used to build a gene catalogue. The advent of NGS technologies, including protocols for mRNA sequencing (RNA-seq), permits a rapid generation of transcriptomes for any species [3]. The main advantages of transcriptome sequencing are that transcriptome sequences can be obtained rapidly, the lower cost comparing to the sequencing of a complete genome, and the ability to estimate expression abundances. However, this approximation has also several limitations. Transcripts lack the key regulatory sequences of interest of a gene and several transcripts will be absent in the gene set if they are not expressed in the sampled tissue or are not sequenced at sufficient depth to permit representation in the assembly. Moreover, the analysis of these data sets present several analytical and computational challenges, such as to accurately assemble the short reads from organisms that do not have any reference genome [22]. In any case, bioinformatic databases for the comparison and analysis of this kind of data, similar to that created to compare genomic sequences, have to be developed yet.

## 5. Summary and Outlook

The implementation of second generation sequencing technologies has allowed the completion and the starting of many plant genome projects, mainly in crop species. The continuous development of these methods and the strengthening of third generation sequencing technologies promise to accelerate the achievement of any plant genomic sequence. This rapid increase in available genome sequences is producing an enormous volume of raw information that needs to be processed in order to extract information about gene family architecture and evolution. Thus, new plant-specific comparative genomic databases have been developed. Based on new clustering techniques, comparative genomic databases have become more accurate tools for genome-wide gene family classification and for the prediction of new protein families. The development of these databases and the implementation of novel methods will be crucial to infer gene families and orthologous genes in the near future, as well as to integrate published functional data into comparative genomic databases and to re-evaluate the accuracy of the gene families detected.
